# Hematological changes in anemic dairy calves treated with a hematinic complex

**DOI:** 10.14202/vetworld.2025.994-1001

**Published:** 2025-04-25

**Authors:** Roberto González-Garduño, Fleider Leiser Peña-Escalona, Rocío Hernández-Díaz, Carlos Luna-Palomera, Ema de Jesús Maldonado-Siman, Ever del Jesus Flores-Santiago, Alfonso J Chay-Canul

**Affiliations:** 1 South-Southeast Regional University Unit, Autonomous University of Chapingo, Teapa, Tabasco, Mexico; 2 Bachelor’s Degree in Business Administration and Management, Polytechnic University of Texcoco, Estado de Mexico. Mexico; 3 Department of Engineering, Technological Institute of the Altiplano of Tlaxcala, National Technology of Mexico, San Diego, Xocoyucan, Tlaxcala, Mexico; 4 Academic Division of Agricultural Sciences, Juarez Autonomous University of Tabasco, Villahermosa, Tabasco, Mexico; 5 Postgraduate in Animal Production, Autonomous University of Chapingo, Texcoco, Estado de Mexico. Mexico

**Keywords:** anemia, calves, haematocrit, haemoglobin, hematinic complex, red blood cells, tropical cattle systems

## Abstract

**Background and Aim:**

Weaning is a critical period in calf development, particularly under tropical conditions where nutritional stress and parasitic infestations can precipitate anemia. Anemia compromises growth and survival; yet, few studies have evaluated the effectiveness of hematinic therapy in anemic calves before weaning in tropical systems. This study aimed to assess hematological responses in anemic dairy calves treated with a hematinic complex and to establish anemia thresholds based on hematological indices in healthy calves raised under tropical conditions.

**Materials and Methods:**

A total of 22 Holstein × Zebu calves were studied from January to April 2024 in Tabasco, Mexico. Calves were grouped as healthy (n = 10; hematocrit [HCT] >24%) or anemic (n = 12; HCT <24%). The anemic group received an intramuscular hematinic complex for 5 consecutive days and three additional doses at day 42. Hematological parameters were measured every 21 days using an automated analyzer. Anemia thresholds were defined from the healthy group using mean ± 2 standard deviations. Data were analyzed using a repeated-measures design over time.

**Results:**

Anemia thresholds were determined as HCT <21.3%, hemoglobin (HGB) <7.5 g/dL, and red blood cell (RBC) <5.4 × 10^6^/µL. At baseline, anemic calves exhibited significantly lower HCT (21.4%), HGB (6.9 g/dL), and RBC (5.9 × 10^6^/µL) compared to healthy controls (p < 0.01). Three calves presented with microcytic hypochromic anemia and one with macrocytic anemia. By day 42, hematinic-treated calves surpassed the anemia threshold, reaching an HCT of 25%, and further increased to 30% following the second treatment. HGB and RBC levels also improved, showing no significant differences from healthy calves at study end. Males responded more robustly to treatment than females.

**Conclusion:**

The administration of a hematinic complex effectively restored hematological parameters in anemic calves within 42 days, with sustained improvement following a second application. This intervention is recommended as part of pre-weaning health protocols to mitigate anemia-related growth setbacks in tropical cattle systems.

## INTRODUCTION

When calves are weaned, changes in their diet result in a substantial reduction in nutrient intake. Furthermore, internal and external parasitic infestations can contribute to the development of anemia in these animals. The hematological profile serves as a key physiological indicator for diagnosing, monitoring, and predicting disease progression, as well as for assessing livestock health and overall productivity [1]. In dairy cattle, anemia, commonly attributed to iron deficiency [2] is defined as a reduction in the blood’s oxygen-carrying capacity. In ruminants, as in other species, it is characterized by decreased values in red blood cell (RBC) indices. Specifically, anemia in cattle is indicated by a hematocrit (HCT) or packed cell volume of <24%, an RBC count of fewer than 5 × 10^6^ cells/µL, and a hemoglobin (HGB) concentration below 8 g/dL [3, 4].

Anemia may result from various causes, including parasitic diseases such as gastrointestinal nematode (GIN) infections. Hematophagous species such as *Haemonchus contortus*, *Haemonchus placei* [5], and *Mecistocirrus digitatus* [6] are particularly significant. In addition to anemia, these parasites cause other clinical signs, including pale mucous membranes, dehydration, dark and watery feces, reduced appetite, and weight loss. Their blood-feeding activity induces iron-deficiency anemia and hypoproteinemia in affected hosts [7]. These consequences can substantially reduce average daily weight gain and, in severe cases, result in the death of infected animals [8].

Other significant parasites that cause anemia include tick-borne hemoparasites [9], which are responsible for diseases such as anaplasmosis, babesiosis, and theileriosis [10]. These conditions remain prevalent in tropical regions. Members of the family *Anaplasmataceae* (order *Rickettsiales*), including the genera *Anaplasma* and *Ehrlichia*, are tick-borne pathogens that cause progressive anemia, jaundice, and fever in the absence of hemoglobinuria [11]. Babesiosis, another tick-transmitted disease, is caused by protozoa of the genus *Babesia* (*Babesia bigemina*, *Babesia divergens*, *Babesia bovis*, and *Babesia major*) and is characterized by hemolytic anemia and fever, often accompanied by hemoglobinuria. This disease is globally distributed, affecting numerous mammalian species, with significant impacts on cattle health and productivity [12, 13]. Theileriosis, also a protozoan tick-borne disease affecting ruminants, is caused by *Theileria parva* and *Theileria annulata*, the most pathogenic species in Africa. It leads to East Coast fever, a condition marked by the enlargement of superficial lymph nodes and persistent fever [9, 14].

In addition to parasitic causes, nutritional deficiencies constitute a major contributor to anemia in ruminants. Deficiencies in essential vitamins and minerals, such as cobalt and copper, can impair RBC production. Cobalt is converted by the ruminal microflora into vitamin B12, a crucial cofactor for various enzymatic systems involved in erythropoiesis [3]. Copper is necessary for the mobilization of iron from hepatic stores and its subsequent transport to the bone marrow for RBC synthesis [7].

The influence of seasonal variation on hematological parameters in cattle has been extensively documented, and such data are used to evaluate cattle’s resilience to diverse climatic conditions [1]. Environmental factors, including ambient temperature and relative humidity, are associated with livestock growth and productivity [15]. Moreover, the physiological stage of the animal influences its response to heat stress and, consequently, affects hematological values that are closely linked to performance [16]. Management practices also play a key role in the development of anemia, with weaning representing a particularly critical phase in calf development.

Although anemia in calves is a well-recognized health issue, particularly under tropical production systems, most existing studies have focused on neonatal calves or generalized thresholds derived from non-tropical regions. Limited research has specifically addressed anemia occurring during the weaning phase, a critical period marked by dietary changes and increased exposure to parasites. Furthermore, there is a scarcity of data on hematological reference values for diagnosing anemia in pre-weaned calves reared under humid tropical conditions. In particular, the therapeutic potential of hematinic complexes to reverse anemia and improve hematological parameters during this vulnerable stage remains underexplored in tropical cattle production systems.

This study aimed to evaluate the hematological response to a hematinic complex in anemic dairy calves raised under tropical conditions. Specifically, the objectives were to (1) establish threshold values for RBC indices to diagnose anemia based on healthy calf profiles, (2) characterize the type and severity of anemia in weaned calves, and (3) assess the efficacy of a hematinic treatment in restoring hematological parameters over time. The findings are intended to support the development of evidence-based health management strategies for calves in tropical environments.

## MATERIALS AND METHODS

### Ethical approval

This study was approved by the General Directorate of Research and Postgraduate Studies of the Autonomous University of Chapingo (project 24027-C-67), in accordance with the Mexican Official Standard NOM-051-ZOO-1995 for the humane treatment and mobilization of animals.

### Study period and location

The study was conducted from January to April 2024, during which forage availability was limited due to lower temperatures in January and reduced rainfall from March to April. The study was conducted at the experimental farm of the Southeast Regional University Unit, Autonomous University of Chapingo, located in Teapa, Tabasco, Mexico (17°31’36.1”N, 92°55’49.7”W). The region has a warm and humid climate, with an average annual temperature of 26.6°C and annual precipitation of 3085 mm [17].

### Animal management

A total of 22 Holstein × Zebu calves were included in the study. Animals were allocated into two groups based on their hematological status, particularly HCT levels. Calves with HCT levels >24% were designated as healthy, whereas those with HCT <24% were classified as anemic. The healthy group (n = 10) had a mean age of 6.3 ± 2.4 months and a mean weight of 89.8 ± 24.1 kg, while the anemic group (n = 12) had a mean age of 7.9 ± 2.8 months and weighed 86.2 ± 21.5 kg at baseline. Group allocation was non-random and based on convenience, which may limit the study’s repeatability due to reliance on the natural occurrence of anemia, itself influenced by livestock management practices.

Anemia in these calves developed following weaning and grazing on *Urochloa decumbens* pastures. Management changes, along with tick infestation and GIN presence, contributed to weight loss and health deterioration. One calf died from anemia and was excluded from the study. Post-initial sampling, male calves were confined, while females continued grazing. Anemic calves received a hematinic product (Hemoplex; Laboratorios Andoci S.A., Mexico City), with the following composition per 100 mL: sodium cacodylate, 3 g; iron citrate, 2 g; cobalt acetate, 50 mg; copper sulfate, 50 mg; manganese chloride, 5 mg; and Vitamin B12, 50 µg. Each calf received 5 mL intramuscularly daily for 5 consecutive days, followed by three additional doses 42 days later.

After initial sampling, all calves were treated for GIN using levamisole phosphate 22.3% (Fosfamisol^®^ M.V., Biogénesis Bagó, Argentina) and treated externally for tick control using amitraz 12.5% (Bovitraz, Bayer de México S.A. de C.V., México DF). To meet their nutritional requirements, calves were fed a formulated diet consisting of 2% oil palm, 40% pelleted forage, 35.9% ground corn, 1% wheat bran, 2% molasses, 16% soybean meal, 1% urea, and 2% mineral mix. Confined calves received 5 kg of this feed daily, supplying 14% crude protein and 2.96 Mcal/kg of digestible energy to support growth [18].

### Parasitological sampling

At the onset of the study, fecal samples were collected to determine GIN egg counts using the McMaster technique [19]. Positive samples were subjected to copro-culture, and infective larvae were recovered after 8 days and morphologically iden-tified [20]. All animals were subsequently dewormed with Fosfamisol^®^ M.V. at the commercial dose of 8 mg/kg body weight. Follow-up fecal sampling was conducted on day 21 post-treatment to confirm parasite clearance.

### Hematological sampling

To monitor hematological responses, whole blood samples were collected every 3 weeks from the jugular vein using ethylenediaminetetraacetic acid-containing vacutainer tubes (Vacutainer^™^; BD Biosciences, Franklin Lakes, NJ, USA). Samples were analyzed using a fully automated hematology analyzer calibrated for bovine use (MTD H51; MTD Diagnostics S.R.L., San Nicola la Estrada, Italy), which provided a complete blood count, including RBC and platelet indices.

Anemia thresholds for RBC parameters were determined using hematological data from healthy calves (n = 10; total samples = 50), calculated as the mean ± 2 standard deviations (SD) to establish 95% confidence limits. These thresholds were used to classify calves as anemic based on HCT and RBC counts. HGB values (HGB ± 2 SD) were used to classify anemia by color intensity (hypochromic, normochromic, hyperchromic), while mean corpuscular volume (MCV ± 2 SD) was used to define cell size categories (microcytic, normocytic, and macrocytic) [21].

### Statistical analysis

Hematological variables (RBC and platelet parameters) were analyzed using a repeated measures design over time in two treatment groups: healthy and anemic calves. Statistical analysis was performed using the Statistical Analysis System software package version 9.4 [22], applying the following model:

Y_ijk_=µ+τ_i_+δ_j(i)_+P^k^+(τP)^ik^+ε_ijk_

where: Y_ijk_ represents the response variable (hematological variables). µ is the overall mean; τ_i_ denotes the effect of the treatment (i.e., anemic or healthy); δ_j(i)_ is the random error associated with the animal (subject) within the treatment; P_k_ indicates the effect of the period (with values at 1, 12, 21, 42, 70, and 81); (τP)_ik_ represents the treatment-by-period interaction; and ε_ijk_ is the random error associated with the repeated measurement within the animal.

## RESULTS

### Parasitological response

At the onset of the study, anemic calves exhibited a fecal nematode egg count of 250 ± 190 eggs/g of feces, whereas healthy calves had only 13 ± 25 eggs/g. The predominant species identified in the coprocultures were *Cooperia punctata* (84%), *Oesophagostomum* spp. (11%), and *Mecistocirrus digitatus* (5%). The presence of these blood-feeding nematodes, along with horn flies (*Hematobia irritans*) and ticks (*Rhipicephalus microplus*), likely contributed to the development of anemia. Additi-onally, the dietary transition associated with weaning and subsequent grazing could have exacerbated the condition. In contrast, the healthy calves, though also grazing, continued nursing and did not develop anemia.

### Determination of anemia thresholds

Anemia thresholds were established using hematological data from healthy calves, defined as the mean minus two SD. The values determined were: HCT < 21.3%, HGB < 7.5 g/dL, and RBC count < 5.4 × 10^6^/µL. For classifying anemia type based on RBC size, the mean cell volume (MCV) range was set at 28.5–45.3 femtoliters (fL). To assess color intensity, the HGB range used was 7.5–12.7 g/dL (Table 1).

**Table 1:** Average values of hematological parameters of the red formula of healthy *Holstein-Zebu* calves (10 calves, 50 records) and thresholds.

Parameter	Mean	SD	Lower limit (Threshold) mean − 2 SD	Upper limit mean + 2 SD
RBC (106/µL)	8.4	1.5	5.4	11.4
HGB (g/dL)	10.1	1.3	7.5	12.6
HCT (%)	30.5	4.6	21.3	39.8
MCV (fL)	36.9	4.2	28.5	45.3
MCH (pg)	12.2	1.3	9.6	14.7
MCHC (g/dL)	33.1	1.5	30.1	36.2
RDW (%)	21.1	1.8	17.4	24.8
PLT (10^3^/µL)	393.9	108.7	176.6	611.2
MPV (fL)	6.1	0.5	5.1	7.2

RBC=Red blood cell count, HGB=Hemoglobin, HCT=Hematocrit, MCV=Corpuscular volume, MCH=Corpuscular hemoglobin, MCHC=Corpuscular hemoglobin concentration, RDW=Red blood cell distribution width, PLT=Platelets, MPV=Mean platelet volume, SD=Standard deviation, fL=Femtoliters

At baseline, significant differences were noted between the two groups (p < 0.01). Anemic calves had an average HCT of 21.4%, RBC count of 5.9 × 10^6^/µL, and HGB of 6.9 g/dL, while healthy calves showed values of 34.9%, 9.6 × 10^6^/µL, and 11.2 g/dL, respectively (Table 2).

**Table 2:** Hematological parameters of anemic calves compared to healthy calves.

Parameter	Healthy group (control)		Anemic group (treated)	
	
	Mean	SD	Mean	SD
Number	10	-	12	-
RBC (10^6^/µL)	9.6^a^	1.2	5.9^b^	1.4
HGB (g/dL)	11.2^a^	0.9	6.9^b^	1.6
HCT (%)	34.9^a^	3.8	21.4^b^	4.8
MCV (fL)	36.7^a^	3.2	36.6^a^	2.9
MCH (pg)	11.7^a^	0.7	11.8^a^	0.8
MCHC (g/dL)	32.1^a^	1.4	32.3^a^	0.7
RDW (%)	20.7^a^	0.9	20.8^a^	2.9
PLT (10^3^/µL)	349.9^b^	62.0	478.9^a^	185.0
MPV (fL)	6.3^a^	0.3	6.3^a^	0.4

Different letters (a, b) in each row represent significant differences (p < 0.05). RBC=Red blood cell count, HGB=Hemoglobin, HCT=Hematocrit, MCV=Corpuscular volume, MCH=Corpuscular hemoglobin, MCHC=Corpuscular hemoglobin concentration, RDW=Red blood cell distribution width, PLT=Platelets, MPV=Mean platelet volume, SD=Standard deviation, fL=Femtoliters

In the first sampling, seven calves met the criteria for anemia, and five additional calves with HCT values near 24% were included in the anemic group. Only one calf displayed macrocytic anemia (MCV > 45.3 fL), while three calves exhibited microcytic anemia, detected within the first two samplings (i.e., the initial 14 days). Based on HGB levels, all cases of anemia were classified as hypochromic, with values below 7.5 g/dL (Table 3).

**Table 3:** Type of anemia based on the thresholds constructed with the group of healthy calves.

ID	Day	Sex	Age (months)	RBC (10^6^/µL)	HGB (g/dL)	HCT (%)	MCV (fL)	RDW (%)	Size	Density
Q2	1	Female	10.3	6.52	7.4	23.7	36.5	22.0	Normocytic	Hypochromic
Q3	1	Female	10.3	5.44	7.3	23.1	46.2	17.5	Macrocytic	Hypochromic
Q4	1	Male	10.1	4.77	5.7	18.1	38.0	18.1	Normocytic	Hypochromic
Q6	1	Male	9.9	3.51	4.4	13.6	39.0	19.1	Normocytic	Hypochromic
Q6	12	Male	10.3	4.05	4.3	13.1	32.5	23.0	Normocytic	Hypochromic
Q6	21	Male	10.6	5.22	5.4	17.2	33.1	22.5	Normocytic	Hypochromic
Q7	1	Female	9.9	4.93	5.6	17.2	35.0	18.0	Normocytic	Hypochromic
Q7	12	Female	10.3	6.09	6.3	19.7	32.4	21.3	Microcytic	Hypochromic
Q9	1	Male	9.6	4.3	4.5	13.8	32.1	21.5	Microcytic	Hypochromic
Q9	12	Male	10.0	6.11	4.9	16.3	26.7	25.8	Microcytic	Hypochromic
Q16	1	Female	8.6	5.51	6.3	19.3	35.2	19.6	Normocytic	Hypochromic

RBC=Red blood cell counts, HGB=Hemoglobin, HCT=Hematocrit, mean cellular volume, RDW=Red blood cell distribution width, MCV=Corpuscular volume, fL=Femtoliters

**Figure 1:**
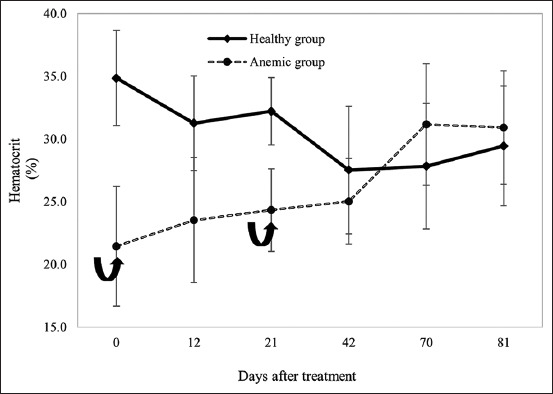
Evaluation of hematocrit in hematinic-complexed calves compared with untreated calves. D0: Five applications of the hematinic complex. D42: Three applications of the hematinic complex.

During the treatment phase, anemic calves demonstrated a progressive increase in HCT following hematinic administration. By day 42, HCT values surpassed the anemia threshold, reaching 25%. After a second round of treatment, HCT further increased to 30%, and no statistically significant differences (p > 0.05) were observed between the anemic and healthy groups (Figure 1).

**Figure 2:**
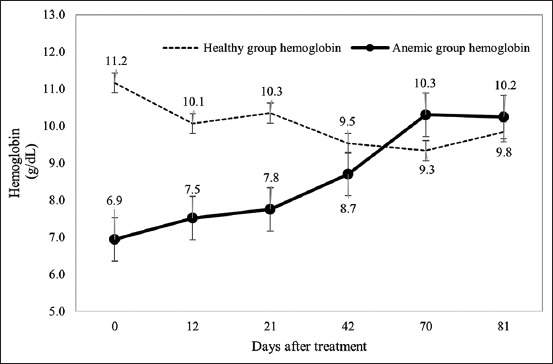
Evaluation of hemoglobin concentration in calves treated with a hematinic complex compared with untreated calves. D0: Five applications of the hematinic complex. D42: Three applications of the hematinic complex.

HGB levels followed a similar trajectory. A significant increase in HGB was observed over time in the treated anemic calves (p < 0.01), reaching values comparable to those of the healthy group by day 42 (Figure 2).

### Treatment and sex

Among anemic males, HCT rose markedly by 45.1%, from 17.4% to 25.3%, within the first 42 days (p < 0.01). After the second hematinic administration, HCT increased by an additional 28.9%, reaching 32.6% at 70 days. In contrast, anemic females, which remained on pasture, showed a more modest response: An initial 6.2% increase (from 23.5% to 24.9%) and a subsequent 21.9% increase (from 24.9% to 30.4%) following the second treatment.

**Figure 3:**
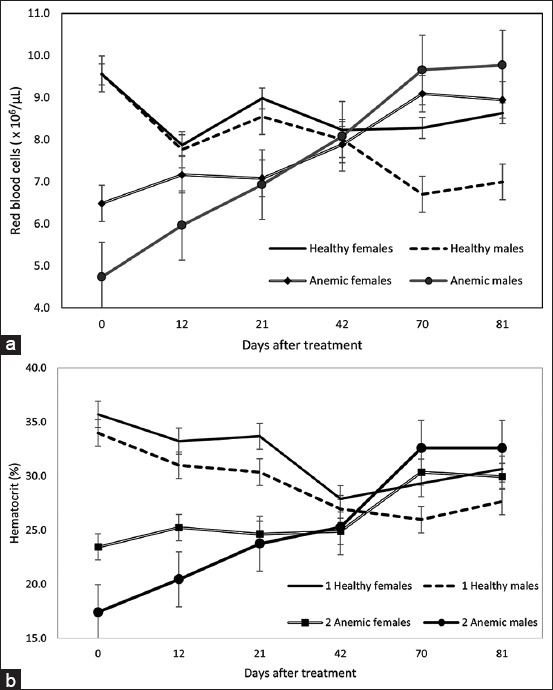
(a) Red blood cell counts by sex and days post-treatment. (b) Hematocrit changes in the anemic and healthy groups by sex according to days elapsed after the first treatment.

Among the healthy calves (both males and females), a decline in HCT and RBC counts was observed, likely due to age-related physiological changes. One calf exhibited an unexpected, pronounced drop in HCT during the final two samplings. By the conclusion of the study, the mean HCT in the anemic group exceeded that of the healthy group; however, the difference was not statistically significant (Figure 3).

## DISCUSSION

### Weaning and health challenges in extensive livestock systems

Weaning in extensive livestock farming is a practice that exposes calves to nutritional, social, physical, and psychological stressors, resulting in alterations to their physiological, behavioral, and health responses [23]. In southeastern Mexico, calves are typically weaned at 7 or 8 months of age, weighing between 140 kg and 160 kg [24]. Similar weaning practices are observed in other tropical regions, such as Venezuela, where calves are weaned at approximately 8 months [25]. Despite efforts to provide nutritional support before weaning, calves still undergo stress-induced changes that can lead to reduced body weight, disease [26], and anemia, often triggered by behavioral responses associated with weaning. This study highlights the recovery of health in calves suffering from severe anemia induced by weaning under tropical conditions – an issue frequently encountered on farms where pre-weaning management is generally inadequate.

### Thresholds for determining anemia

Although the diagnosis of anemia has been extensively examined in neonatal calves, iron suppleme-ntation is commonly recommended as a prev-entive measure [2, 8, 27]. However, many studies have not defined anemia in terms of severity thresholds, underscoring the importance of establishing such criteria for tropical regions to better understand the magnitude of the problem. A prior study conducted in the humid tropics of Mexico reported confidence intervals for hematological parameters indicative of anemia in calves as follows: HCT <20.1%, HGB <6.1 g/dL, and RBC count <5.9 × 10^6^ cells/µL [4]. These values are comparable to those found in the present study, despite a smaller sample size (n = 50). In both cases, anemia thresholds were calculated as the mean minus 2 SD [4].

The reference values identified in this study align with those reported in other sources [28, 29]. However, many of these references do not distinguish between calves reared in tropical environments and those raised under different conditions, often applying generalized values. For instance, one study defined anemia in ruminants as a packed cell volume <24%, RBC count <5 × 10^6^ cells/µL, or HGB <8 g/dL [29]. While some widely cited sources classify anemia based on an HCT of 21% [28], this threshold is intended for adult cows rather than growing calves. Nevertheless, this value has been adopted by other researchers as a general reference for anemia in young animals [3].

Several studies have proposed acceptable reference ranges, but few define anemia explicitly or include statistical criteria for establishing confidence limits [30, 31]. For example, one study presented an HGB range of 8.4–12 g/dL, which is too high to serve as a diagnostic threshold for anemia [32]. Another study utilized HGB as an indirect indicator of iron status, setting the lower threshold at 72.5 g/L, which was considered deficient [2]. The reference intervals for RBC count reported by three different sources were 4.9–7.5, 5–10, and 5.1–7.6 × 10^6^ cells/µL—ranges that closely match the lower limit established in this study (5.4 × 10^6^ cells/µL). Similarly, reported HCT ranges vary between 21%–30%, 28%–38%, and 22%–30%, supporting the adoption of 21.3% as an appropriate lower threshold for diagnosing anemia in calves [32]. In this study, the threshold for anemia in healthy calves was set at 21.3%, which is consistent with general reference standards. Although some studies have classified anemia severity based on HCT values, categorizing no anemia (24%–26%), mild (20%–23.99%), moderate (12%–19.99%), and severe (<12%) [33, 34], such classification was not applied here.

### Erythrocyte morphology and anemia type

In the present study, most anemic calves exhibited microcytic anemia, with MCV values below 28.5 fL. This contrasts with findings from another study that reported macrocytic anemia in 16 calves, with MCV values as high as 100 fL [35]. In our research, only one case of macrocytic anemia was observed, in which the MCV slightly exceeded the threshold of 45.3 fL (recorded at 46.2 fL). A study from South Korea reported MCV values ranging from 52 to 56 fL, likely reflecting the larger erythrocyte size in adult cattle compared to young animals [34]. The MCV values reported here are consistent with those indicated in *Veterinary Hematology* [28] for cows, which range from 36 to 50 fL, affirming the relevance of our findings for calves.

### Recovery of hematological parameters

The anemia observed in this study was hemolytic in nature, a regenerative condition wherein the bone marrow compensates for RBC loss by increasing erythropoiesis and releasing reticulocytes into circulation. Consequently, RBC levels rise during regenerative anemia [28]. This case likely involved mild RBC hemolysis, contributing to the increase in HCT levels [34]. The long-term benefits of iron supplementation were evident in the improvement of HGB and HCT values, consistent with other findings where iron dextran-treated calves achieved HGB levels of 11.0 g/dL after 6 weeks, compared to 9.5 g/dL in control animals [2]. The HGB values reported in this study also resemble those documented in African calves [36], where HGB levels ranged from 9 to 10 g/dL and HCT values remained below 30%, with confidence intervals approximating the anemia threshold of 21%.

The hematological improvements observed reinforce the benefits of iron therapy, particularly in enhancing HCT levels [8]. In one study using a t-test, no significant differences were found in hematological parameters between male and female anemic calves [33]. One limitation of the present study is the difficulty in identifying sufficient numbers of anemic animals, which constrained the statistical power of treatment-by-sex interactions. Nevertheless, the primary treatment effects are considered reliable due to the low variability in hematological data.

## CONCLUSION

This study demonstrated that anemia in weaned dairy calves raised under tropical conditions can be effectively identified and managed using hematinic supplementation. Anemia thresholds were established using hematological data from healthy calves, with HCT <21.3%, HGB <7.5 g/dL, and RBC <5.4 × 10^6^/µL, providing a valuable diagnostic reference tailored to tropical rearing systems. The administration of a hematinic complex significantly improved hematological parameters in anemic calves, with HCT levels surpassing the anemia threshold within 42 days and continuing to rise after a second round of treatment. Notably, male calves housed in confinement exhibited a more pronounced response compared to grazing females.

A key strength of this study lies in its field-based design, which reflects practical conditions faced by farmers in tropical environments. The results provide evidence-based recommendations for managing anemia in calves during the critical weaning phase. In addition, the use of locally adapted hematological thresholds enhances the relevance of the findings to similar production systems in humid and subtropical regions.

However, the study also has limitations. The non-randomized group assignment and small sample size, particularly in treatment-by-sex interactions, may restrict the generalizability of the findings. Furthermore, the identification of naturally occurring anemia limited replication and control across experimental groups.

Future research should aim to validate the proposed anemia thresholds across different tropical regions and production systems. Integrated weaning protocols that include nutritional conditioning, parasite control, and timely hematinic supplementation should be developed and evaluated. Moreover, future studies should explore the long-term effects of anemia correction on growth performance, immune status, and productivity in calves. These efforts are essential to improve animal welfare, health outcomes, and economic returns in tropical livestock production.

## AUTHORS’ CONTRIBUTIONS

RGG: Conceptualization, data curation, formal analysis, and investigation. FLPE: Data curation and drafted the manuscript. RHD, CLP, and EJFS: Validation visualization and drafted, reviewed, and edited the manuscript. EJMS: Formal analysis and interpretation and reviewed and edited the manuscript. AJCC: Project administration, validation, and reviewed and edited the manuscript. All authors have read and approved the final manuscript.
